# Adaptation and Validation of the 3 × 2 Achievement Goals Questionnaire in a Population of Athletes

**DOI:** 10.3390/bs14040350

**Published:** 2024-04-22

**Authors:** Cristina García-Romero, Elkin Eduardo Roldan-Aguilar, Carlos Alberto Hurtado-Castaño, Josune Rodríguez-Negro, Oliver Ramos-Álvarez

**Affiliations:** 1 Faculty of Education, Camilo José Cela University, 28692 Madrid, Spain; crisgr30@gmail.com; 2 Isabel I University, 09003 Burgos, Spain; 3 Semillero de Investigación en Fisiología del Fisiología del Ejercicio, Politécnico Colombiano Jaime Isaza Cadavid, Medellín 050022, Colombia; eeroldan@elpoli.edu.co (E.E.R.-A.); carlosahurtado@elpoli.edu.co (C.A.H.-C.); 4 Department of Didactics of Musical, Plastic and Corporal Expression, Faculty of Education, University of the Basque Country (UPV/EHU), 48940 Leioa, Spain; josune.rodriguez@ehu.eus; 5 Research Unit of School Sport, Physical Education and Psychomotricity of the University of A Coruña, 15008 A Coruña, Spain; 6 Health Economics and Health Services Management Research Group, Marqués de Valdecilla Research Institute (IDIVAL), 39011 Santander, Spain; 7 Department of Education, Physical and Sports Education Area, University of Cantabria, Los Castros Avenue, 50, 39005 Santander, Spain; 8 Facultad de Ciencias de la Salud, Universidad Europea del Atlántico, 39011 Santander, Spain; 9 Facultad de Ciencias Sociales, Universidad Europea del Atlántico, 39011 Santander, Spain

**Keywords:** motivation, 3 × 2 achievement goals, validation, life satisfaction, sport

## Abstract

(1) Background: Sport goals, although widely recognised as crucial for motivation and performance in sport, are multifaceted and can be difficult to measure directly. The present research aims to validate the 3 × 2 achievement goals questionnaire of Mascret in Spanish in a population of athletes. (2) Method: By using a latent factor approach, it is possible to identify the underlying dimensions of these goals and to better understand how they are structured. For this purpose, this questionnaire has been translated and compared with the life satisfaction scale. An exploration of the multifaceted nature of sport goals has been carried out using structural equation modelling. A total of 580 athletes (463 males and 216 females, M = 21.5, SD = 2.36) from different sport disciplines and from 12 autonomous communities in Spain participated in the research. (3) Results: The results show that the questionnaire presents a high scale reliability and that all items contribute significantly to the internal consistency of the scale. (4) Conclusions: The adaptation of this scale to the Spanish population of athletes can be a valid and useful tool to measure and understand motivation and goals in the sport context.

## 1. Introduction

Over the last 30 years, achievement goals have been a reference in motivational research in the context of sport and physical activity. This theory evidences that achievement goals are the reasons that people direct their behaviour towards goals; i.e., individuals’ goals lie in striving to demonstrate competence and ability in achievement contexts [1].

The literature on achievement goals has progressed according to an evidenced structure: researchers come up with a conceptual model, theorists develop a measure to assess that model, and finally, the instrument is implemented to analyse its development in different contexts, from which it is extracted whether the model is valid and functional for assessing competence. This procedure has forged a wide network of studies on the understanding of achievement goals [2,3,4,5,6].

The concept of achievement goals emerged in the early 1980s and differentiated between two goals: mastery and performance [1]. In this so-called dichotomous model, mastery goals were oriented towards developing competence through mastery and improvement of tasks, whereas performance goals were interpreted as striving to demonstrate competence in relation to others [7]. Research on the dichotomous model focused on a variety of settings, including school [7,8] and sport [9].

Following research in this field in the 1990s, and after questioning the model both empirically and conceptually, it was proposed to extend the model by incorporating approach–avoidance valences [3]. This model, called the trichotomous model, consisted of the mastery goal (similar to the dichotomous model), the performance-approach goals (focused on doing well in relation to others), and the performance-avoidance goals (focused on not doing badly in relation to others). In this particular model, several measures emerged, for school, work, and sport contexts [10,11,12].

Years later, Elliot and McGregor (2001) [4] expressed that mastery-based and performance-based goals could be bifurcated, differentiating between the two types of competence definition (mastery and performance) and the two types of competence valence (approach and avoidance). Therefore, the model consisted of four achievement goals, being called the 2 × 2 model: the mastery-approach goals (the traditional mastery goal), the mastery-avoidance goals (focused on not doing badly in relation to the demands of the task), the performance-approach goals (classic performance goal; tries to achieve interpersonal competence), and the performance-avoidance goals (based on avoiding doing worse than others). This measure was tested in different settings, including school [4,13,14] and sports [5,15].

The last model that the achievement goal theory yields, the so-called 3 × 2 model, presents three standards for assessing competence: task, self, and other. Task-based goals use the absolute demand of the task as a reference for assessment; self-based goals use one’s own trajectory for competence assessment; and other-based goals are the most complex to adopt, as they assess competence in relation to doing well or poorly in relation to other peers [16]. The model therefore consisted of six achievement goals: the task-approach goals (focused on achieving task-based competence), the task-avoidance goals (focused on avoiding task-based incompetence), the self-approach goals (focused on self-based competence), the self-avoidance goals (focused on avoiding doing worse than how one performed in the past), the other-approach goals (focused on achieving competence based on others), and the other-avoidance goals (focused on avoiding doing worse than one’s peers). The instrument of this model was applied in the school context [16,17]; in the French sport context, a validation was conducted by Mascret et al. (2015) [6].

Numerous reasons are evident for carrying out the validation of the 3 × 2 achievement goals questionnaire with a focus on sport in the Spanish language. The first of these is the conceptual aspect, as it is relevant to differentiate between task-based and self-based standards in the sport domain, i.e., to assess whether athletes participating in a sport or physical activity can focus on whether or not they are achieving as a function of the task itself, or how they are doing in relation to how they did in the past or in their future. Empirically, current measures of mastery-approach and mastery-avoidance vary in different terms depending on whether they focus on task-based standards [18], self-based standards [5], or a combination of task-based and self-based standards [19].

The aim of the present study is to adapt, validate, and test the psychometric property of the 3 × 2 achievement goals questionnaire in the sport context in a sample of Spanish athletes.

## 2. Materials and Methods

### 2.1. Participants

The sample consisted of 580 athletes (463 males and 216 females, M = 21.5, SD = 2; figure skating, Pilates, powerlifting, rowing, rugby, triathlon, volleyball, and water polo) from 12 autonomous communities in Spain (Asturias, Castilla-La Mancha, Madrid, Cantabria, Andalusia, Valencia, Basque Country, Castilla y León, Murcia, Catalonia, Aragon, and the Balearic Islands). The athletes completed both questionnaires anonymously in their training sessions.

### 2.2. Instruments

Achievement Goals in Sport Questionnaire (CML 3 × 2-D): The questionnaire was validated in Spanish based on the one developed by Mascret et al. (2015) [6]. The items were preceded by “In sport my goal is…”. This instrument is composed of a total of 18 items grouped into six factors: task-approach (e.g., “…to obtain good results”), task-avoidance (e.g., “…to avoid having bad results”), self-approach (e.g., “…. do better than in the past”), self-avoidance (e.g., “…avoid doing worse than I usually do”), other-approach (e.g., “…do better than others”), and other-avoidance (e.g., “…avoid doing worse than others”). Cronbach’s alphas in the Mascret et al. (2015) [6] study ranged from 0.93 (other-avoidance) to 0.80 (task-approach). Participants indicated the degree of agreement with each of these statements using a 7-point Likert scale, ranging from 1 (strongly disagree) to 5 (strongly agree).

### 2.3. Procedure

The questionnaire was based on the scale of Mascret et al. (2015) [6] and is composed of 18 items grouped into 6 factors. The questionnaire was sent to the sample participating in the research to be completed during their training sessions. Once all the data had been collected, the relevant statistical analyses were carried out for its validation.

### 2.4. Statistical Analysis

A confirmatory factor analysis was conducted using the R-4.3.3 software package (Lucent Technologies, Alpharetta, GA, USA). A three-factor model was fitted to the questionnaire responses, which consisted of statements related to sport goals, such as “In sport my goal is to perform well” and “In sport my goal is to avoid performing poorly”. These items were used to infer three latent factors, interpreted as “personal performance goals”, “competitive goals”, and “efficacy goals”.

Confirmatory factor analysis (CFA) was conducted to assess the structure of the sport goals defined by the variables ML1 to ML18. The theoretical model defined three latent factors: MR1, reflecting goals related to personal performance; MR2, representing competitive goals; and MR3, describing goals related to efficacy.

The proposed model included a total of 129 parameters and was fitted to data from 850 observations using the Diagonally Weighted Least Squares (DWLS) estimator. Convergence was achieved after 41 interactions.

## 3. Results

**Figure 1:**
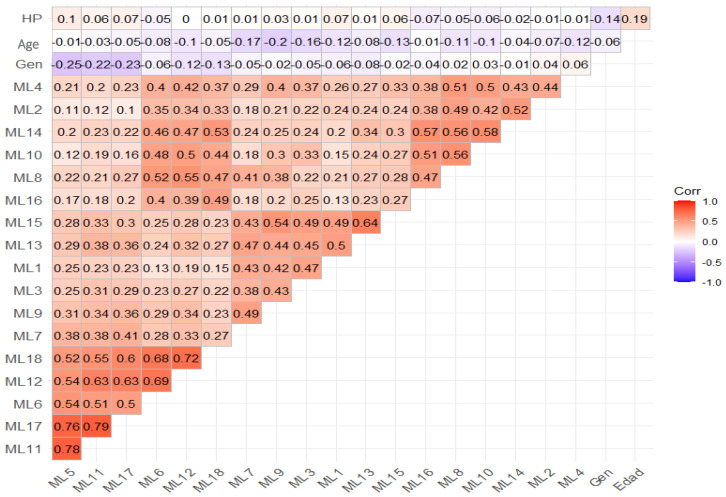
Correlation chart between items.

Although the aim of this research is confirmatory, it was considered relevant to assess the exploratory results in order to justify the number of latent factors. For this reason, a review of the correlation between the variables was initially carried out, in which the correlations between the different items assessed in the population can be seen (Figure 1).

**Figure 2:**
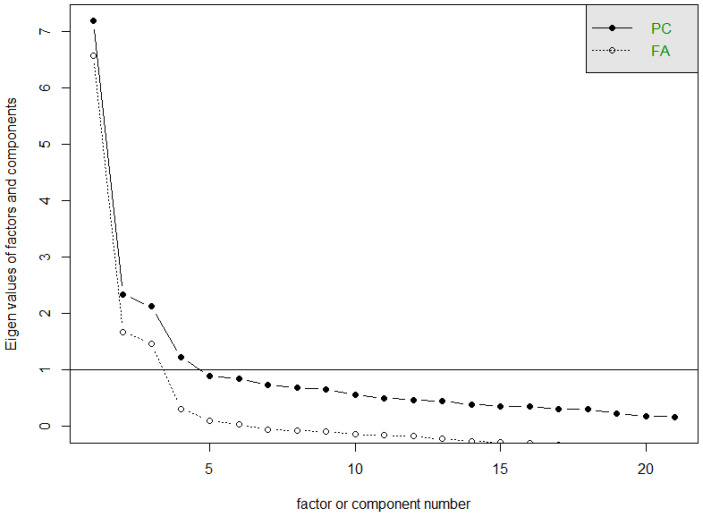
Sedimentation graph.

**Figure 3:**
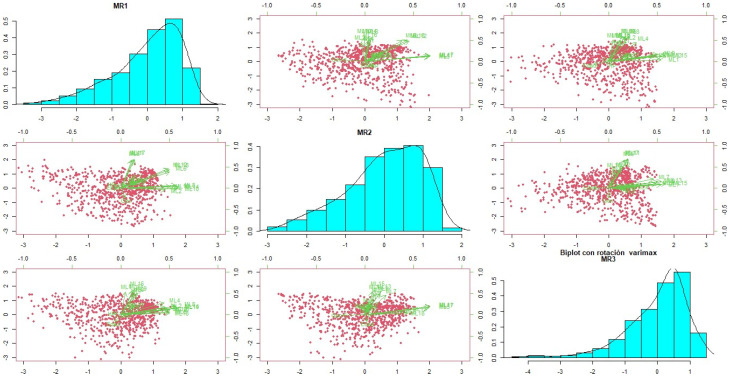
Biplot graph with Varimax rotation.

Of these correlations, while low correlations are expected, the higher-order correlations presented here refer to three groupings that are detailed in the process of constructing latent factors confirmed in the sedimentation plot and the biplot (Figure 2 and Figure 3).

As can be seen from Table 1, the chi-square test of the model was significant (χ^2 = 2056.834, df = 132, *p* < 0.001), indicating that the proposed model does not exactly reproduce the observed covariance matrix. However, the chi-square test is sensitive to sample size and can be significant even if the discrepancy between the model and the data is trivial. Therefore, other fit indices were also considered.

From the same table, it can be seen that the values of the Comparative Fit Index (CFI) and the Tucker–Lewis Index (TLI), which are less sensitive to sample size, were 0.974 and 0.970, respectively. These values are very close to 1, suggesting a good fit of the model to the data. However, the Root Mean Square Error of Approximation (RMSEA) was 0.131, which exceeds the recommended threshold of 0.08 for a good fit. This indicates that the model has an average discrepancy per degree of freedom that is larger than is generally considered acceptable.

The RMSEA is a measure of model fit that takes into account the complexity of the model. Smaller values (usually less than 0.06) indicate a better fit. Here, both the standard and robust RMSEA are above this threshold (0.131 and 0.151), suggesting that the model can be improved.

The SRMR is a measure of the average difference between the observed correlations and the correlations predicted by the model. Values less than 0.08 are generally considered acceptable. In this case, the SRMR is 0.095, which is close to but above the recommended threshold.

Therefore, while some of the model fit metrics seem to suggest that the model fits the data reasonably well, others indicate that there may be room for improvement in the model. In particular, it may be useful to investigate further to identify potential problems with the model, such as variables that do not fit well or complex relationships between variables that are not captured in the current model.

In terms of the variables under study and the latent factors found, these results imply that, although the three latent factors appear to represent distinct aspects of participants’ goals in sport, the model may not fully capture the correlation between the different goals.

The discrepancy in the fit indices suggests that, although the model may capture a large proportion of the variance in the data (as indicated by the CFI and TLI), there may also be model specifications that do not fit the data perfectly. These results highlight the importance of using multiple fit indices and considering the results of the CFA in the context of past and future analyses. Table 2 provides the estimation and interpretations of the latent factors in the model.

The characteristics of the three goals analysed can be distinguished, and are as follows:

- Personal performance goals (MR1): This factor includes items that reflect a motivation to improve personal performance as well as to avoid underperformance. Individuals with high scores on this factor may be particularly focused on their personal sport performance, regardless of the performance of others.
- Competitive goals (MR2): This factor reflects the motivation to compete and outperform others in sport performance. Individuals with high scores on this factor may be especially competitive and oriented to outperform their peers.
- Efficacy goals (MR3): This factor is composed of items that refer to being effective and improving effectiveness over time. Individuals with high scores on this factor may be especially motivated to be effective in their sport and improve their effectiveness over time.

Table 3 shows the estimates of the covariances between the latent factors (MR1, MR2, and MR3). The estimated values represent the estimated covariance between each pair of factors, with a higher value indicating a higher relationship. Standard errors (Std.Err) represent the uncertainty in the covariance estimates.

The z-value is a hypothesis test that indicates whether each covariance estimate is significantly different from zero. Large (in absolute terms) z-values and small P(>∣z∣) values (all 0.000 in this case) provide strong evidence against the null hypothesis that the covariance equals zero. That is, these results indicate that each pair of latent factors has a significant relationship.

The Std.lv value refers to the standardised covariance between the latent factors, indicating the strength of the relationship between them. This shows that the three latent factors (MR1, MR2, and MR3) are significantly related to each other. The strongest covariance is observed between MR1 and MR2, followed by the covariance between MR1 and MR3, and finally the covariance between MR2 and MR3. This suggests that the goals in sport related to these factors are intrinsically linked.

### 3.1. Confirmatory Factor Analysis

**Figure 4:**
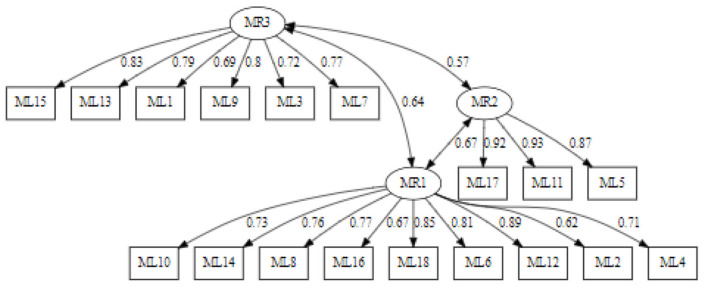
Lavaan chart.

The Lavaan plot depicted in Figure 4 shows the factor loadings (the relationships between the latent variables and the observed variables) as well as the correlations between the latent factors, which show how these factors are related to each other.

### 3.2. Reliability Analysis of the Achievement Goals Test: Dep

The results are presented in Table 4.

The reliability of the Sport Goals Test scale was assessed using Cronbach’s alpha (α), a measure of internal consistency. The calculation of α is based on the number of items (k) and the ratio of the summed item variances to the total variance of the scale. Formally, α can be represented as

$$\alpha =\frac{k}{k-1}\left(1-\frac{\sum_{i=1}^{k}\sigma_{Yi}^{2}}{\sigma_{X}^{2}}\right)$$

where $\sigma_{Yi}^{2}$ is the variance of the i-th item, and $\sigma_{Yi}^{2}$ is the total variance of the total observed scores. The raw α obtained for the Dep scale was 0.78.

The standardised α (0.89) was also calculated. This form of α is based on the assumption that all items share the same variance, and is computed as

$$\alpha_{\mathrm{std}}=\frac{k\bar{r}}{1+\left(k-1\right)\bar{r}}$$

where $\bar{r}$ is the average inter-item correlation.

The average inter-item correlation (0.27) and the median inter-item correlation (0.25) were reported. The signal-to-noise ratio, calculated as the ratio of the variance of the true score to the variance of the error, was 7.9.

The standard error of measurement (SEM) of the scale, which reflects the precision of the scale, was also calculated. The SEM is calculated as the standard deviation of the scale scores multiplied by the square root of (1 − α), resulting in a value of 0.011 for the Dep scale.

The reliability of the scale was further assessed using Feldt’s confidence interval and Duhachek and Iacobucci’s confidence interval, which indicated a range between 0.76 and 0.8 for the α at the 95% confidence level.

Regarding item-to-item reliability, i.e., quantifying how much each item contributes to the overall consistency of the data set, Table 5 can be observed.

It is notable that, without exception, the alpha calculated for each of the variables inserted into the model exceeded 0.75, which is generally considered acceptable or good, suggesting that the items or scale questions are highly related to each other and therefore likely to measure the same underlying construct [20]. Specifically, a Cronbach’s alpha of 0.75 indicates that the construct is reliably measured with relatively high internal consistency. This provides evidence of the reliability of the measure, which is an important aspect of its validity.

In a confirmatory factor analysis (CFA) model, this suggests that the model has identified a coherent underlying latent factor and that the scale items cluster effectively around this factor.

In order to confirm the results presented, the statistics presented in Table 6 were calculated.

From Table 6, it can be noted that G.6 is also a measure of scale reliability and is considered more robust than Cronbach’s alpha, especially when item response distributions are not normal. In this case, the value of G.6 is 0.92, which suggests a high level of scale reliability. On the other hand and from the same table, the omega hierarchical indicates the variance that can be explained by a general factor in a set of items. A value of 0.57 indicates that approximately 57% of the variance of the items can be explained by a general factor, suggesting that a general factor underlies most of the items.

Omega H asymptotic is an estimate of the maximum amount of variance that can be attributed to a general factor, based on the upper bound of the variance explained by that factor. A value of 0.62 suggests that up to 62% of the variance of the items could be explained by a general factor.

And finally, omega total represents the total proportion of item variance that can be attributed to all common factors, both general and specific. A value of 0.92 indicates that most of the variance can be explained by the common factors, which is an indication of high reliability.

**Figure 5:**
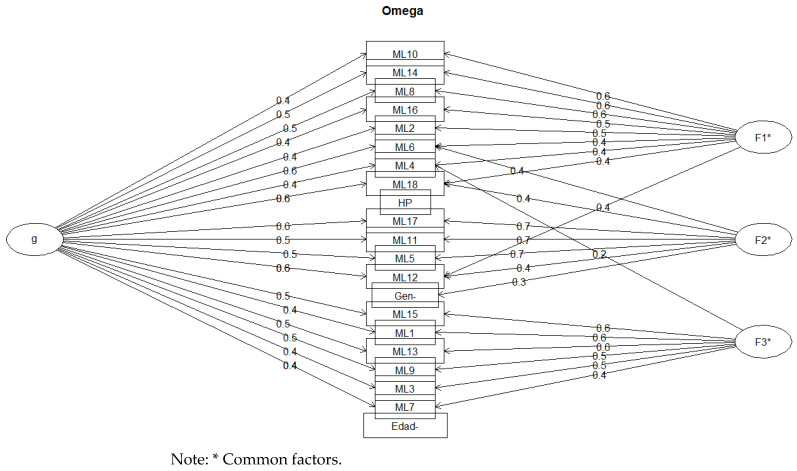
Graphical representation of the structural CFA model.

These results, as well as the calculation of the effects of each variable on each of the estimated underlying factors, can be seen in Figure 5.

## 4. Discussion

The questionnaire data show that the results of the reliability tests (Cronbach’s alpha and McDonald’s omega) suggest that the set of items shows high internal consistency, with a Cronbach’s alpha of 0.88–0.89 and an omega total of 0.92–0.93. These values above 0.7 indicate that the items consistently measure the same latent construct, providing strong evidence of the scale’s reliability; these data are congruent with studies conducted by Mascret et al. (2017) [21] in a sample of teachers, and Ning (2018) [22] and Kadioglu-Akbulut and Uzuntiryaki-Kondakci (2019) [23] in the Hong Kong and Turkish populations, respectively.

With reference to the impact of item deletion, it can also be concluded that the results of the reliability analysis if one item is deleted indicate that all items contribute significantly to the internal consistency of the scale. The removal of any of the items would result in a decrease in Cronbach’s alpha or McDonald’s omega, so it is suggested to keep all items in the scale.

The data extracted from the study indicate that task- and self-directed goals are distinct goals in the sport context, as Mascret et al. (2015) [6] showed that competence was positively correlated with task-directed goals but was not related to self-goals. The previous authors found problems with some of the stated items, especially with the task-approach and task-avoidance item, regarding their wording, which can sometimes appear ambiguous to the respondent, as one can interpret these items as self- or other-based. Since the domain of sports involves techniques, skills, and strategies, items assessing task goals should involve these three dimensions.

Inter-item correlations show high relationships between items from the other-avoidance goal, whereas inter-construct correlations are highest between task-approach and self-goals and task-avoidance and self-goals, these data being consistent with the studies of Mascret et al. (2015) [6], Cecchini et al. (2021) [24], and García-Romero et al. (2022) [25].

Studies on the achievement goal theory showed that mastery goals were related to positive outcomes, such as high levels of academic achievement and self-efficacy [26,27,28], personal well-being, relationship building and intrinsic and identified motivation [29], self-determined motivation [30], and autonomy [31]. In contrast, the relationships for the adoption of performance goals were not clearly defined. Some researchers found that these goals were associated with negative and maladaptive outcomes, such as anxiety [32] and burnout [29], because their central focus revolves around outperforming others [33]. However, other studies did not support this hypothesis, as in some achievement contexts, performance goals showed null results, mainly due to their temporally unstable nature [29].

## 5. Conclusions

Each extension of the dichotomous model adds precision to the achievement goal theory, leading to the current 3 × 2 model. This shows, as indicated in the review by Lochbaum et al. (2023) [34], that the data from the 3 × 2 achievement goals questionnaires are more strongly supported in the sport context than in the education context, with large differences in the significance of the task and other competencies.

Finally, it is important to highlight that, although the scale shows a high degree of reliability, further studies should be conducted to validate it in different contexts and populations. Furthermore, the conclusions obtained so far speak of the scale’s reliability, but further research is still needed to examine its content, criterion, and construct validity.

The results obtained so far are promising and suggest that the scale may be a useful tool for measuring and understanding motivation and goals in the sport context.

## Figures and Tables

**Table 1:** Model fit indices.

Fit Index	Value	Acceptable Fit
Chi-square	2056.834	>0.05
Degrees of freedom	132	-
CFI	0.974	>0.90
TLI	0.970	>0.90
RMSEA	0.131	<0.08
SRMR	0.095	<0.08

Note: CFI: Comparative Fit Index; TLI: Tucker–Lewis Index; RMSEA: Root Mean Square Error of Approximation; SRMR: average difference between the observed correlations and the correlations predicted by the model.

**Table 2:** Parameter estimates for latent variables.

	Estimate	Std.Err	z-Value	*P*(>|*z*|)	Std.lv	Std.all
**MR1**						
ML10	1.000	-	-	-	0.727	0.727
ML14	1.049	0.027	39.185	0.000	0.762	0.762
ML8	1.062	0.025	41.870	0.000	0.771	0.771
ML16	0.915	0.028	32.427	0.000	0.665	0.665
ML18	1.164	0.028	41.439	0.000	0.846	0.846
ML6	1.117	0.028	40.177	0.000	0.811	0.811
ML12	1.225	0.027	44.790	0.000	0.890	0.890
ML2	0.856	0.032	26.541	0.000	0.622	0.622
ML4	0.977	0.029	33.415	0.000	0.710	0.710
**MR2**						
ML17	1.000	-	-	-	0.924	0.924
ML11	1.002	0.011	90.474	0.000	0.926	0.926
ML5	0.940	0.012	81.133	0.000	0.869	0.869
**MR3**						
ML15	1.000	-	-	-	0.829	0.829
ML13	0.955	0.029	33.475	0.000	0.792	0.792
ML1	0.831	0.033	25.421	0.000	0.689	0.689
ML9	0.966	0.029	33.107	0.000	0.801	0.801
ML3	0.870	0.033	26.428	0.000	0.721	0.721
ML7	0.927	0.030	30.867	0.000	0.769	0.769

Note: Std.lv: standardised covariance; Std.Err: standard errors; MR1: personal performance goals; MR2: competitive goals; MR3: efficiency goals.

**Table 3:** Covariances of latent factors in the CFA model.

	Estimate	Std.Err	z-Value	*P*(>|*z*|)	Std.lv
MR1 MR2	0.449	0.017	26.309	0.000	0.669
MR1 MR3	0.386	0.019	20.243	0.000	0.640
MR2 MR3	0.439	0.024	18.328	0.000	0.573

Note: Std.lv: standardised covariance; Std.Err: standard errors.

**Table 4:** Reliability analysis.

Raw_Alpha	Std.Alpha	G6 (smc)	Average_r	S/N	Ase	Mean	Sd	Median_r
0.78	0.89	0.92	0.27	7.9	0.011	9.3	1	0.25
		**lower**		**alpha**		**upper**		
Feldt		0.76		0.78		0.8		
Duhachek		0.76		0.78		0.8		

**Table 5:** Reliability if an item is removed.

Item	Raw_Alpha	Std.Alpha	G6 (smc)	Average_r	S/N	Alpha se	Var.r	Med.r
Age-	0.87	0.89	0.92	0.30	8.4	0.0063	0.035	0.27
Gen-	0.78	0.89	0.93	0.30	8.4	0.0115	0.035	0.27
HP	0.81	0.90	0.93	0.30	8.6	0.0100	0.032	0.27
ML1	0.77	0.88	0.92	0.28	7.6	0.0118	0.039	0.27
ML2	0.77	0.88	0.92	0.28	7.6	0.0122	0.038	0.26
ML3	0.77	0.88	0.92	0.27	7.5	0.0119	0.039	0.25
ML4	0.76	0.88	0.92	0.27	7.4	0.0123	0.038	0.24
ML5	0.76	0.88	0.91	0.27	7.4	0.0123	0.036	0.25
ML6	0.76	0.88	0.91	0.27	7.2	0.0128	0.036	0.25
ML7	0.77	0.88	0.92	0.27	7.4	0.0120	0.039	0.25
ML8	0.76	0.88	0.91	0.27	7.3	0.0126	0.037	0.25
ML9	0.77	0.88	0.92	0.27	7.4	0.0121	0.039	0.24
ML10	0.76	0.88	0.91	0.27	7.4	0.0124	0.037	0.25
ML11	0.76	0.88	0.91	0.27	7.3	0.0124	0.036	0.25
ML12	0.75	0.88	0.91	0.26	7.1	0.0130	0.035	0.24
ML13	0.77	0.88	0.91	0.27	7.4	0.0120	0.038	0.25
ML14	0.76	0.88	0.91	0.27	7.4	0.0125	0.037	0.25
ML15	0.77	0.88	0.91	0.27	7.4	0.0120	0.038	0.24
ML16	0.77	0.88	0.92	0.27	7.6	0.0122	0.038	0.25
ML17	0.76	0.88	0.91	0.27	7.3	0.0125	0.036	0.25
ML18	0.76	0.88	0.91	0.26	7.2	0.0128	0.036	0.25

**Table 6:** Other reliability statistics.

Statistic	Value
Cronbach’s alpha	0.89
G.6	0.92
Omega hierarchical	0.57
Omega H asymptotic	0.62
Omega total	0.92

## Data Availability

Data are contained within the article.
